# Full-length RNA sequencing reveals the mechanisms by which an TSWV–HCRV complex suppresses plant basal resistance

**DOI:** 10.3389/fpls.2023.1108552

**Published:** 2023-03-22

**Authors:** Min Gui, Huaran Hu, Zhiqiang Jia, Xue Gao, Hongzheng Tao, Yongzhong Li, Yating Liu

**Affiliations:** ^1^ College of Plant Protection, Yunnan Agricultural University, Kunming, China; ^2^ Horticultural Research Institute, Yunnan Academy of Agricultural Science, Kunming, China; ^3^ College of Life Science and Technology, Honghe University, Mengzi, China; ^4^ College of Tobacco, Yunnan Agricultural University, Kunming, China

**Keywords:** *tomato spotted wilt orthotospovirus* (TSWV), Hippeastrum chlorotic ringspot orthotospovirus (HCRV), co-infection mechanism, basal resistance, full-length transcriptome, miRNA

## Abstract

Viruses deploy numerous strategies to infect plants, typically by forming complexes with another virus, leading to more efficient infection. However, the detailed plant responses to viral infection and the underlying mechanisms of co-infection remain unclear. Previously, we found that *tomato spotted wilt orthotospovirus* (TSWV) and Hippeastrum chlorotic ringspot orthotospovirus (HCRV) could infect plants in the field by forming a complex. In this study, we found that TSWV infected tobacco (*Nicotiana benthamiana*) plants in cooperation with HCRV, leading to a more efficient infection rate of both viruses. We then used the in-depth full-length transcriptome to analyze the responses of *N. benthamiana* to complex infection by TSWV–HCRV (TH). We found that infection with individual TSWV and HCRV triggered plant defense responses, including the jasmonic acid signaling pathway, autophagy, and secondary metabolism. However, TH co-infection could not trigger and even suppress some genes that are involved in these basal resistance responses, suggesting that co-infection is advantageous for the virus and not for the plants. Typically, the TH complex inhibits *NbPR1* expression to suppress tobacco resistance. Moreover, the TH complex could alter the expression of microRNAs (miRNAs), especially novel-m0782-3p and miR1992-3p, which directly interact with *NbSAM* and *NbWRKY6* and suppress their expression in tobacco, leading to downregulation of *NbPR1* and loss of resistance in tobacco to TSWV and HCRV viruses. Overall, our results elucidated the co-infection mechanisms of TH in tobacco by deploying the miRNA of plants to suppress plant basal resistance and contributed to developing a novel strategy to control crop disease caused by this virus complex.

## Introduction

Hippeastrum chlorotic ringspot orthotospovirus (HCRV) (Dong et al., 2013) and *tomato spotted wilt orthotospovirus* (TSWV) are members of the Tospoviridae family (Lefkowitz et al., 2018). Both viruses can infect a range of plants, particularly solanaceous crops, which leads to leaf malformation, local chlorosis, and necrosis, and then a serious reduction in crop production and subsequent economic losses (Dong et al., 2013; Li et al., 2017). TSWV has been listed as one of the top 10 plant viruses in the world, which causes annual losses of more than 1 billion USD (Qi et al., 2021). Pesticides are widely used to control viral infections in agriculture, but their misuse and overuse can lead to environmental pollution and still cannot fundamentally control these diseases induced by TSWV and HCRV. Breeding resistant cultivars is still the most effective strategy for controlling this disease. However, the lack of understanding of the TSWV/HCRV–plant interaction has limited the deployment of disease-resistant germplasm resources.

During the process due to interactions with pathogenic viruses, plants have evolved a variety of defensive systems, particularly the defense responses regulated by hormones, transcription factors, and mitogen-activated protein kinases (MAPKs) (Jones et al., 2006; Pieterse et al., 2009; Dodds et al., 2010; Meng and Zhang, 2013; Calil et al., 2017; He et al., 2020; Mauck et al., 2020; Zhou and Zhang, 2020). Therefore, understanding plant resistance responses to both viruses will contribute to the breeding of resistant cultivars. MAPKs transmit an external stimulation signal to plant cells by activating downstream kinases and transcription factors, which then amplify the signal cascade to activate the expression of resistance genes (He et al., 2020). Some transcription factors in plants can interact with MAPKs and be phosphorylated by them to regulate the expression of downstream target genes (Asai et al., 2002; Meng and Zhang, 2013). In parallel, transcription factors are also involved in hormone signaling pathways to regulate plant defense responses to viral infections (Spoel et al., 2008; Amorim et al., 2017; Yang et al., 2020). Moreover, the jasmonic acid (JA) signaling pathway can also function in plant defense responses to virus infection (Takaoka et al., 2018; Pan et al., 2021). Additionally, plant secondary metabolism acts as an outcome of the plant defense system to defend against virus infection, such as the metabolism of terpenoids, flavonoids, and anthocyanins (Holopainen and Gershenzon, 2010). Thus, plant basal resistance genes provide a mass of potential resistance resources to breed resistant cultivars.

Commonly, plants are infected with multiple or complex infections of different viruses during their growth. It has been reported that multiple viruses, including SMV and BPMV, have a synergistic effect upon host infection to effectively inhibit host plant resistance, resulting in more severe symptoms than infection with either virus alone (Dapalma et al., 2010). Complex infection by AMV and WCMV will promote their successful infection in the field (Xiang, 2013). The mass of the two viruses in the co-infected host was 5,897- and 3,515-fold higher than that in the single-infected host (Liang et al., 2017). Studies have shown that plant viruses can change the nutritional conditions and defense responses of host plants, particularly when complex viruses infect. The expression of several defense-related genes in plants decreased after the combined infection of viruses (Schwarte and Tiedemann, 2011). The ratio of sugar and amino acids in the leaves and phloem of zucchini (*Cucurbita pepo*) decreased significantly after cucumber mosaic virus (CMV) infection, resulting in a reduction in plant resistance (Mauck et al., 2010 and Mauck et al., 2014). The combined infection of tomato yellow leaf curl China virus (TYLCCNV) and its satellite can significantly inhibit the JA defense pathways, leading to decreases in plant resistance (Zhang et al., 2012). Currently, the development of high-throughput sequencing technology has become an effective tool to develop molecular markers and has been widely used in plant genetics and breeding, germplasm conservation, and development. Many studies have been conducted at the transcriptional level using RNA-Seq technology to study plant–pathogen interactions (Naidoo et al., 2018).

In this study, we used in-depth whole-length RNA-Seq sequencing to analyze the different responses of *Nicotiana benthamiana* to complex infections with TSWV and HCRV. TH complex infections differ from individual infections of TSWV or HCRV and can synergistically suppress plant resistance, particularly MAPKs, secondary metabolism, and the JA signaling pathway. Importantly, the TH complex could alter the expression of microRNAs (miRNAs) in tobacco to negatively regulate the plant defense responses associated with individual TSWV and HCRV and then promote their successful infection in plant tissue. In summary, our research provides insight into the defensive responses of plants to complex viral infections, which contributes to the development of an effective strategy to control diseases induced by a virus complex.

## Materials and methods

### Virus inoculation

TSWV (isolate: GC-1) was isolated from lettuce (*Lactuca sativa*) in Yunnan Chenggong, and HCRV (isolate: HLS1-2) was isolated from susceptible spider lily (*Hymenocallis littoralis*) in Kunming. TSWV or HCRV was inoculated and maintained in *Emilia sonchifolia* (L.) plants. Six- to eight-week-old plants were used for viral inoculation. TSWV or HCRV-infected *E. sonchifolia* leaves were collected and ground in an inoculation buffer to inoculate *N. benthamiana* plants. Tobacco (*N. benthamiana*) seedlings with four to five true leaves were divided into four groups, which included inoculation with TSWV, HCRV, and TSWV + HCRV. Buffer inoculation was used as the control group (CK). The inoculum was prepared by grinding 1 g of systemically infected fresh leaves in 10 ml of inoculation buffer (Ocampo et al., 2016). The identified symptomatic young leaves were collected and ground in a sterilized mortar and then dipped in cotton swabs on the tobacco leaves for inoculation. The symptoms were recorded after inoculation at 7 days post-inoculation (dpi). The leaves above the inoculated leaves were collected at 1, 7, and 14 dpi and stored at −80°C.

### RNA extraction, strand-specific library construction, and sequencing

Total RNA from different treatments of tobacco with three biological replicates was extracted using a TRIzol reagent kit (Invitrogen, Carlsbad, CA, USA) according to the manufacturer’s instructions. The rRNAs were then removed to retain the mRNAs and non-coding RNAs (ncRNAs). The enriched mRNAs and ncRNAs were fragmented into short fragments using fragmentation buffer and reverse transcribed into cDNA with random primers. Next, the cDNA fragments were purified with a QIAquick PCR Extraction Kit (Qiagen, Venlo, Netherlands), end-repaired, poly(A) added, and ligated to Illumina (San Diego, CA, USA) sequencing adapters. The digested products were sequenced using Illumina HiSeq™ 4000 by Gene Denovo Biotechnology Co. (Guangzhou, China).

### RT-qPCR assay

Total cDNA was prepared from RNA using the Superscript III™ Reverse Transcriptase kit (Invitrogen, Cat. 18080–044). A one-tenth dilution of the reverse transcription final reaction was prepared; 1 μl of the dilution was used as a template for the qPCR consisting of 0.4 μM of each primer and 1× SYBR Green Master Mix (QuantiTect^®^ SYBR^®^ Green PCR Kit; Qiagen, Cat. 2041453). Transcript levels were normalized to the expression level of *Actin*.

### Data processing

The short read alignment tool Bowtie2v2.4.4 (Langmead and Salzberg, 2012) was used to map reads to a ribosomal RNA (rRNA) database. The reads were further filtered by FASTPv0.23.1 (Chen et al., 2018), so that high-quality clean reads were obtained (Chen et al., 2018). An index of the reference genome was then constructed and paired-end clean reads were mapped to the reference genome using HISAT2v2.1.0 (Kim et al., 2015) with “-rna-strandness RF.” The reconstruction of transcripts was performed with the software StringTiev2.2.0 (Pertea et al., 2016) combined with HISAT2. Novel transcripts were then aligned to the nonredundant (Nr), Kyoto Encyclopedia of Genes and Genomes (KEGG), and Gene Ontology (GO) databases to annotate functional proteins.

### Quantification of transcript abundance and differentially expressed transcripts

Transcript abundances were quantified using the software StringTiev2.2.0 in a reference-based approach. For each transcription region, a fragments per kilobase of transcript per million mapped reads (FPKM) value was calculated to quantify the abundance of expression. The differentially expressed transcripts of coding RNAs and long non-coding RNAs (lncRNAs) were analyzed, respectively. Differential expression analysis of the RNAs and lncRNAs was performed using DESeq2v3.11 (Li and Dewey, 2011) software between the two different groups. The genes/transcripts with the parameter of false discovery rate (*FDR*) below 0.05 and absolute fold change ≥2.0 were considered differentially expressed genes/transcripts.

### RNA ligase-mediated-rapid amplification of cDNA ends (RLM-RACE) assay

The *N. benthamiana* plantlets under normal conditions were used for RNA extraction. The 5’ RLM-RACE adapter was obtained through RNA processing following the instructions in the book. Then, the cDNA acquired by reverse transcription was used as the template for nested PCR according to the protocol of the FirstChoice^®^ RLM-RACE kit (no. AM1700; Invitrogen). The cloned products were purified for further sequencing.

### Vector construction and genetic transformation in tobacco

To construct TRV vectors, 350 bp DNA fragments of target genes of *N. benthamiana* were cloned using a primer pair containing EcoRI and BamHI sites and then ligated to pTRV2. The correct recombinant vectors were transformed into Agrobacterium GV3101, then injected into tobacco. The empty vector-containing Agrobacterium solution was used as a control. At 14 dpi, the transcript level of target genes in plants was assessed using RT-qPCR. The silenced tobaccos were inoculated with HCRV and TSWV.

### Data and enrichment analyses

A principal component analysis (PCA) was performed with the R package gmodels v2.18.1.1 (http://www.r-project.org/). The correlation coefficient between the two replicas was then calculated to evaluate the repeatability between samples based on the Pearson value.

Gene Ontology and KEGG pathway enrichment analyses were performed using the R package Clusterprofilerv4.0 (Wu et al., 2021). GO has three ontologies, which include molecular function, cellular component, and biological process. Significantly enriched GO or KEGG pathway terms in the DEGs that were compared with the genome background were defined by a hypergeometric test. The p-values were calculated using FDR correction and taking *FDR* ≤0.05 as a threshold. The GO enrichment results were visualized using the online tool omicshare (https://www.omicshare.com/), while the scatter plot of KEGG pathway results was generated using ggplot2v3.3.0 (Ito and Murphy, 2013).

MapMan software v3.1 (Thimm et al., 2004) was used to visualize cacao gene expression data in the context of metabolic pathways. MapMan uses a plant-specific ontology that classifies genes into well-defined hierarchical categories, denominated BINs. Cacao genes were assigned to BINs using the Mercator automated annotation pipeline (http://mapman.gabipd.org/web/guest/mercator). Differentially represented MapMan pathways were defined by a two-tailed Wilcoxon rank sum test corrected by the Benjamin–Hochberg method (*FDR* = 0.05).

### Network analysis

The protein–protein interaction network was identified using String v10 (Subramanian et al., 2005) and visualized using Cytoscape software v3.7.2 (Shannon, 2003). In the PPI network, nodes represent the target proteins, while edges represent the predicted or validated interactions between proteins.

The topological network of miRNA and its targets was visualized using Cytoscape v3.7.2 (Shannon, 2003). Topological analysis of target genes was performed using the NetworkAnalyzer plug-in and CytoNCA plug-in of Cytoscape. Target proteins were filtered separately according to betweenness centrality (BC), closeness centrality (CC), and degree centrality (DC), which were calculated using the CytoNCA plug-in. The top 10 miRNAs of each subnetwork were retrieved, and overlapped genes were selected as key targets in the present research.

### miRNA Library construction and sequencing

The total RNA molecules in the size range of 18–30 nt were enriched by polyacrylamide gel electrophoresis (PAGE). The 3’ adapters were then added, and the 36–48 nt RNAs were enriched. The 5’ adapters were then ligated to the RNAs as well. The ligation products were reverse transcribed by PCR amplification, and the 140–160 bp size PCR products were enriched to generate a cDNA library and sequenced by Gene Denovo Biotechnology Co. using Illumina HiSeq X. All the clean tags were aligned with small RNAs in the GenBank database (Release 209.0) to identify and remove rRNA, small coding RNA (scRNA), small nucleolar RNA (snoRNA), small nuclear RNA (snRNA), and tRNA. All the clean tags were also aligned with the reference genome.

### Identification of known miRNA and expression analysis

All the clean tags were then searched against the miRBase database (Release 22) to identify known (species studied) miRNAs. All the unannotated tags were aligned with the reference genome. The level of miRNA expression was calculated and normalized to transcripts per million (TPM). The differential expression of miRNAs was analyzed using edgeR software v4.2 (Robinson et al., 2010) between the two different groups. We identified miRNAs with a fold change of ≥2.0 and *P <*0.05 in a comparison as significant differentially expressed miRNAs. Based on the sequences of the existing miRNAs, known miRNAs, and novel miRNAs, the candidate target genes were predicted using the software PatMatch (Version 1.2).

### Overexpression of miRNAs in tobacco

Full-length miRNAs (Accession numbers: novel-m0693-5p, novel-m0782-3p, novel-m0915-3p, novel-m1488-5p, and novel-m1992-3p) of *N. benthamiana* were cloned using specific primers to construct overexpression vectors. The DNA products were double-digested with EcoRI and BamHI and ligated to the pBIN121-MYC vector restricted by the same enzymes. The correct recombinant vectors were confirmed by sequencing.

Positive colonies of *Agrobacterium* GV3101 harboring overexpression vectors or empty vectors were cultured in 5 ml of LB liquid medium plus 50 mg/L kanamycin and 20 mg/L rifampicin with shaking (200 rpm) at 28°C to an OD 600 value of 2.0, followed by centrifugation at 8,000 rpm for 10 min. The *Agrobacterium* cells were re-suspended to an OD 600 of 0.6 in MES buffer (10 mM MES-KOH, pH 5.2, 10 mM MgCl_2_, and 100 µM acetosyringone). The overexpression of miRNAs in tobacco was performed through agro-infiltration. Then, the expression of resistance genes in tobacco with miRNA overexpression was determined using RT-qPCR, and the experiments were performed three times.

## Results

### Time course transcriptome profiles of *N. benthamiana* following infection with TSWV, HCRV, and TSWV–HCRV

**Figure 1:**
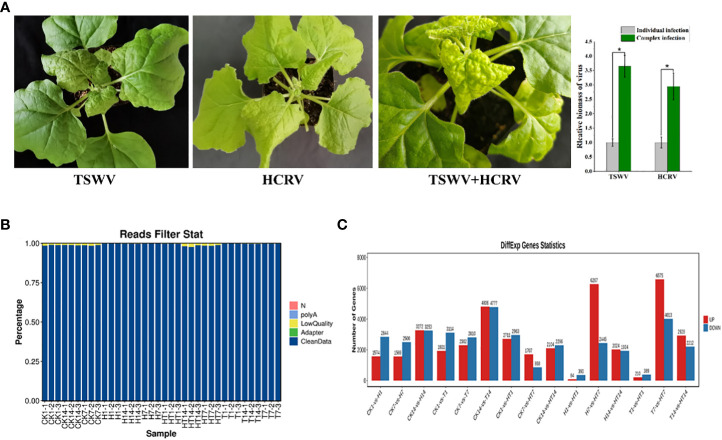
Overview of transcriptome profiles of *Nicotiana benthamiana* under different conditions. **(A)** Symptoms induced by viruses on tobacco seedlings. The biomass of HCRV and TSWV is determined using RT-qPCR. “*: *P <*0.05”. **(B)** The percentages of clean reads in each sample show the high quality of the transcriptome data used in this study. **(C)** The number of differentially expressed genes in each pairwise comparison. The red and blue columns show the significantly up and downregulated genes in each comparison.

In this study, we found that both TSWV and HCRV could infect the leaves of *N. benthamiana*, leading to observable lesions on the related leaves at 7 dpi (**Figure 1A**). Symptoms on tobacco leaves began to appear at 15 days post-inoculation (dpi) of individual HCRV, including chlorosis, deformity, and necrosis of leaves (**Figure 1A**). Meanwhile, a single TSWV inoculation could cause symptoms on tobacco leaves at 7 dpi, and it mainly induced concentric ring lines, chlorosis, yellowing, and necrosis of leaves (**Figure 1A**). Notably, TSWV and HCRV could synergistically infect the tobacco leaves as a complex and induce more observable lesions on the leaves at 7 dpi compared with the individual infection of TSWV or HCRV; the biomass of TSWV and HCRV was the highest in tobacco at 7 dpi of co-infection (**Figure 1A**). The incidence rate of lesions caused by co-infection of the TH complex was faster than that of single HCRV or TSWV (**Figure 1A**). TH complex inoculation mainly caused symptoms on tobacco leaves at 5–7 dpi, and it mainly induced symptoms represented by concentric rings, chlorosis, yellowing, and necrosis of leaves (**Figure 1A**).

Subsequently, the transcriptomes of *N. benthamiana* inoculated with TSWV (T), HCRV (H), and the TSWV–HCRV (TH) complex during different disease response stages (1, 7, and 14 dpi) were sequenced using an Illumina platform. Healthy leaves without infection were used as a control (CK). A total of 264.8 Gb of clean reads were obtained from the 36 samples, which were included in the Illumina sequencing data from all the treatments. After filtering the low-quality reads, polyA, and adaptor, we obtained high-quality clean reads in the transcriptome profiles (**Figure 1B**). All the clean reads from each sample were then mapped uniquely to the *N. benthamiana* reference database. We compared all the samples to identify the significant differentially expressed genes (DEGs) using the threshold *FDR* ≤0.05 and log 2 |fold change| >1 (**Figure 1C**). The results showed that all the TSWV, HCRV, and TH complexes induced dramatic variations in the transcription pattern of *N. benthamiana* (**Figure 1C**). There were 1,574 upregulated and 2,844 downregulated genes in *N. benthamiana* following 1 dpi of HCRV. TSWV induced the differential expression of more genes in *N. benthamiana* at 14 dpi (**Figure 1C**). Consistently, the most significant variations in gene expression caused by TSWV infection were also identified at 14 dpi (**Figure 1C**). Additionally, the variations induced by TH complex infection were smaller than those induced by TSWV and HCRV (**Figure 1C**). Although the expression differences between T1, H1, and TH1 were small, the TH samples exhibited significant changes in gene expression at 7 dpi compared with T and H (**Figure 1C**). Thus, we propose that the HT complex could alter the pattern of expression of *N. benthamiana* to escape the plant defense responses and contribute to their ability to infect.

### Infection of TSWV, HCRV, and TSWV–HCRV complex induced dynamic variations in the pattern of transcription of *N. benthamiana* during the infection and developmental stage

**Figure 2:**
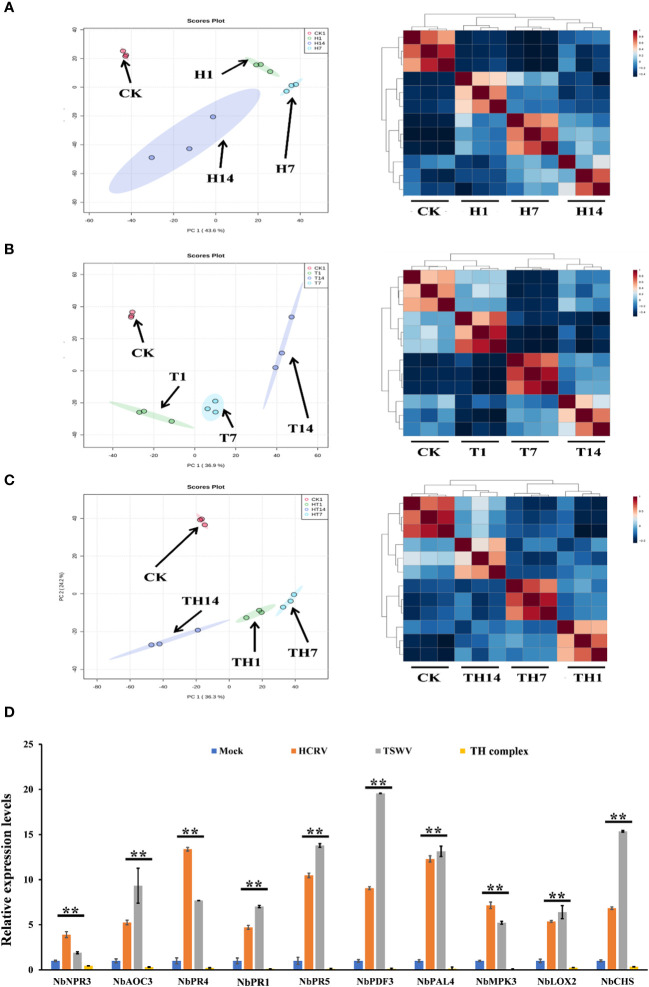
H, T, and TH inoculation caused dramatic variations in the transcriptome of *Nicotiana benthamiana*. Principal component analysis on the transcriptome profiles of tobacco (*N. benthamiana*) at 1-, 7-, and 14-days post-inoculation of H **(A)**, T **(B)**, and TH **(C)**. All the samples are shown in different colors and clustered into one region according to their treatments. The T ellipse represents the 95% confidence interval. Principal components 1 and 2 were used to construct the PCA plots. Unsupervised hierarchical clustering displays the correlation between the samples used in this study and shows four distinct clades, which include three comprised of infected plants post 1-, 7-, and 14-day inoculation of H **(A)**, T **(B)**, and TH **(C)**, and the other of healthy plants. **(D)** RT-qPCR detects the expression levels of DEGs relevant to resistance from transcriptome profiles of *N. benthamiana* following individual H, T, or TH complex inoculation; **P < 0.01.

To investigate the influences of TSWV, HCRV, and TH complex infection on the dynamic transcriptome pattern of *N. benthamiana*, a principal component analysis (PCA) was performed on all the transcriptome profiles from each treatment with three biological replicates (**Figure 2**). We identified that the dynamic expression patterns of tobacco following HCRV infection were significantly affected by TSWV infection during its infection stage, and the samples after 1, 7, or 14 dpi of TSWV were distributed in different regions (**Figure 2A**). Principal component 1 explained 43.6% of the differences among all the samples, and these differences were primarily induced by HCRV infection (**Figure 2A**). Remarkably, the differences in the transcriptome of *N. benthamiana* gradually increased with the time of HCRV infection (**Figure 2A**). The region of samples at 14 dpi of HCRV was separated the most from a region that contained the control samples, indicating that HCRV induced dramatic variations in the transcriptome of *N. benthamiana* at 14 dpi (**Figure 2A**). Further unsupervised hierarchical clustering of samples from the HCRV treatments reached a consensus with the related PCA result, showing that the main differences were generated by HCRV infection at 14 dpi (**Figure 2A**). Moreover, data showed that all the samples under the same treatments closely clustered on one branch, and a comparison of the difference between T14 vs. CK was more significant than that in T1 vs. CK and T7 vs. CK comparisons (**Figure 2A**). Additionally, the primary differences for TSWV infection represented by PC1 explained 36.9% of the variation between all the samples (**Figure 2B**). We found a similar phenomenon from the PCA and unsupervised hierarchical clustering analysis, which supported that the differences in the transcriptome of *N. benthamiana* gradually increased with the time of virus infection, while the primary differences were generated by virus infection at 14 dpi (**Figure 2B**). In contrast, for TH complex infection, the primary differences were generated by infection with the TH complex infection at 1 dpi, and there were 2,711 upregulated genes and 2,963 downregulated genes in the TH1 vs. CK comparison (**Figures 1C**, **2C**). Both PCA and unsupervised hierarchical clustering analysis supported the credibility of our results that all the biological replicates were clustered together and separated from the other treatments (**Figure 2**). Thus, we propose that the TH complex can infect plants in a different manner, which will help both viruses escape plant defense responses.

Subsequently, we performed qPCR assays to validate the gene expression values obtained by RNA-seq. For this purpose, we analyzed the expression of 10 tobacco resistance genes that are triggered by individual virus infection and suppressed by the TH complex (**Figure 2D**). We found that both qPCR assay and RNA-seq results reached a consensus that genes involved in JA-, MAPK-, and transcription factor-mediated defense responses and pathogenesis-related proteins, including *NbPR1*, *NbPR4*, and NbPR5, were triggered to upregulate by individual HCRV or TSWV, while these genes were suppressed to downregulate by the TH complex *in vivo* during the infection process (**Figure 2D**).

### TSWV induced significant changes in plant responses to biotic stress and metabolism

**Figure 3:**
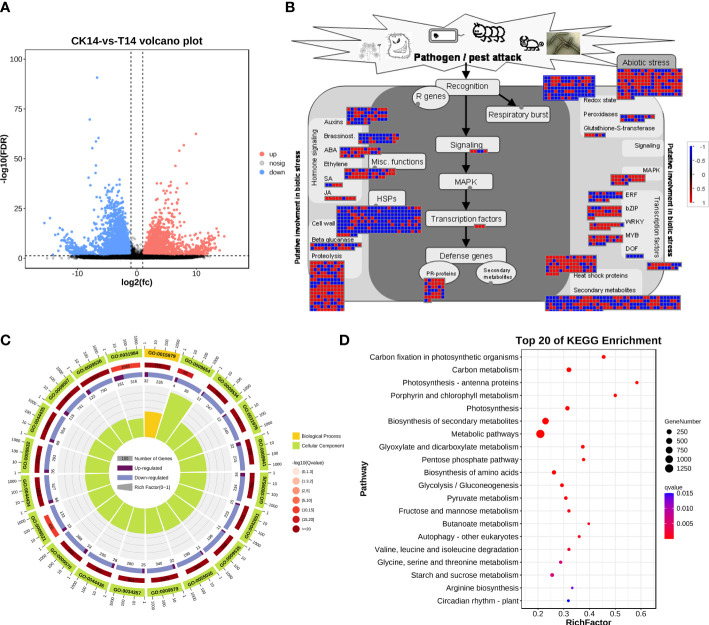
TSWV virus induced dramatic responses relevant to biotic stress in *Nicotiana benthamiana* at 14 days post inoculation. **(A)** Volcano plot of DEGs, which compares the levels of gene expression between CK and T14. Blue dots indicate downregulated genes, and red dots represent upregulated genes. **(B)** MapMan visualization of the biotic responses of *N. benthamiana* to the TSWV virus at 14-day inoculation. Upregulated and downregulated genes are shown in red and blue, respectively. The scale bar represents fold-change values in the CK14 vs. T14 comparison. **(C)** Gene Ontology classification of the DEGs from the CK14 vs. T14 comparison in *N. benthamiana* according to the GO groups based on molecular function, biological process, and cellular component. **(D)** Pathway classification of the DEGs from the CK14 vs. T14 comparison in *N. benthamiana*. The number of genes in each pathway is represented by the plot size. The plot color indicates the significance differences of each pathway.

Considering the main variations in the transcriptome at 14 dpi of TSWV, the transcriptome data of tobacco in the T14 vs. CK comparison was used as a focus to investigate the transcriptomic changes associated with TSWV infection (**Figure 3**). A total of 4,806 upregulated genes and 4,777 downregulated genes were identified as related to TSWV infection (**Figures 1C**, **3A**). We then utilized MapMan software to visualize the involvement of these genes in plant responses to biotic stress (**Figure 3B**). As shown in **Figure 3B**, TSWV infection altered the expression pattern of the genes relevant to plant responses to biotic stress. We noted that TSWV primarily triggered the upregulation of genes involved in MAPK, WRKY, proteolysis, pathogenesis-related (PR) proteins, and secondary metabolism (**Figure 3B**). Most of the genes relevant to MAPK, proteolysis and WRKY were highly expressed in tobacco following TSWV infection at 14 dpi (**Figure 3B**). These genes have been well-characterized in plant defense responses to microbial infection, particularly MAPK, the PR proteins, and secondary metabolism (Pieterse et al., 2009; Zhou and Zhang, 2020). Thus, at 14 dpi, plants could deploy these resistance responses to defend against TSWV infection. Additionally, genes relevant to ethylene synthesis were upregulated in tobacco at 14 dpi, indicating the involvement of ETH in tobacco defense responses against TSWV (**Figure 3B**).

Subsequently, the DEs were collected for KEGG pathway and GO enrichment analyses (**Figures 3C, D**). The significant terms relevant to biological processes, molecular functions, and cellular components from the GO enrichment analysis are shown in **Figure 3C**. Among the terms relevant to biological processes, 306 terms were significantly enriched (*FDR <*0.05; **Table S1**), including photosynthesis, generation of precursor metabolites and energy, organic acid metabolic process, oxoacid metabolic process, carboxylic acid metabolic process, and alpha-amino acid metabolic process (**Figure 3C**; **Table S1**). The most significant terms for cellular components were plastid, chloroplast, thylakoid, chloroplast stroma, photosynthetic membrane, and plastid stroma, suggesting that TSWV infection primarily affected genes that function in plant photosynthesis and nutrient metabolism (**Figure 3C**; **Table S1**). Moreover, most of the genes that were categorized into molecular function terms were involved in cofactor binding, catalytic activity, isomerase activity, coenzyme binding, oxidoreductase activity, and kinase activity (**Figure 3C**; **Table S1**). Additionally, the KEGG pathway enrichment results showed that genes in several pathways were induced, including photosynthesis, biosynthesis of secondary metabolites, alpha-linolenic acid metabolism, and glycerolipid metabolism (**Figure 3C**). Over 80% of all the pathways shown in **Figure 3D** are involved in the plant metabolism of various essential metabolites, such as carbon metabolism, biosynthesis of amino acids, pyruvate metabolism, and glycerolipid metabolism (**Figure 3C**). Alterations in carbohydrate metabolism during the infection of pathogens that enable plants to reduce large amounts of starch granules in infected tissues to mobilize these storage molecules as complementary sources of energy have been well documented (Fan et al., 2010). Furthermore, resistance responses were significantly overrepresented in the DEGs, including secondary metabolism and the α-linoleic acid metabolism pathway associated with JA biosynthesis; most of the related DEGs were upregulated under TSWV infection (**Figures 3B, D**). Thus, we propose that reprogramming of these pathways is involved in the plant–TSWV interaction stage.

### HCRV induced significant changes in the plant responses to biotic stress and metabolism

**Figure 4:**
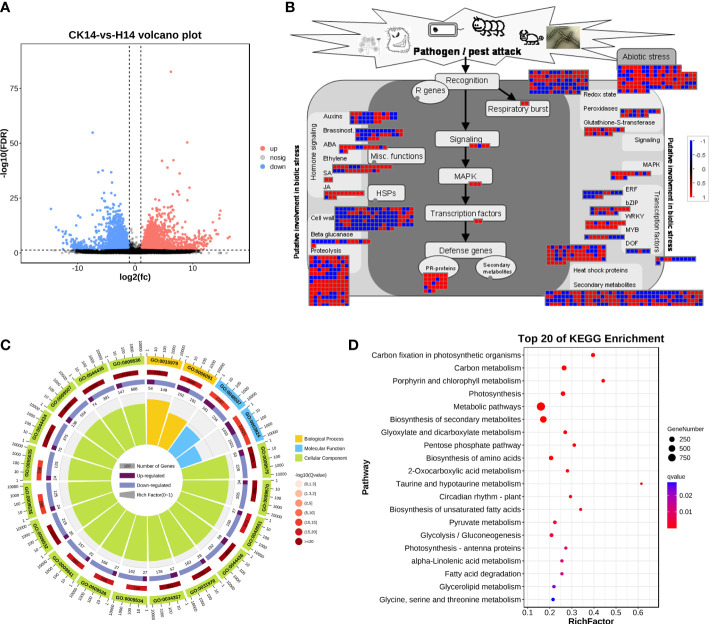
Transcription variations in *Nicotiana benthamiana* at 14 days post inoculation of the H virus. **(A)** Differentially expressed transcripts in *N. benthamiana* at 14 dpi of the H virus. **(B)** Visualization of changes in the expression of genes relevant to the biotic stress of *N. benthamiana* challenged with the H virus. The scale bar represents fold-change values in the CK14 vs. H14 comparison. **(C)** GO analysis of the DEGs in *N. benthamiana* induced by the stress of H virus at 14 dpi. The terms molecular function, biological process, and cellular component are shown. **(D)** KEGG analysis of the DEGs of *N. benthamiana* in the CK14 vs. H14 comparison. The top 20 KEGG enrichment pathways for DEGs are presented. DEGs, differentially expressed genes; dpi, days post inoculation; GO, gene ontology; KEGG, Kyoto Encyclopedia of Genes and Genomes; H, Hippeastrum chlorotic ringspot virus.

For HCRV infection, 3,272 upregulated genes and 3,253 downregulated genes from the H14 vs. CK comparison that responded to HCRV infection were functionally categorized according to Gene Ontology (GO) and KEGG pathway enrichment analyses (**Figure 4**). The GO annotations of DEGs in tobacco following HCRV infection at 14 dpi were enriched for 10,267 GO terms, including 6,622, 2,510, and 1,135 terms relevant to biological process, molecular function, and cellular component, respectively (**Figure 4C**). In the “biological process” domain, the most frequent GO terms were photosynthesis, cofactor metabolic process, small molecule biosynthetic process, vitamin biosynthetic process, carboxylic acid biosynthetic process, carboxylic acid metabolic process, and oxoacid metabolic process (**Figure 4C**; **Table S2**). The results of the cellular component showed that most of the DEGs functioned in the plastid, chloroplast, thylakoid part, and plastid envelope (**Figure 4C**; **Table S2**), suggesting that HCRV also affected plant photosynthesis and essential metabolism. The molecular function GO category indicated that DEGs involved in lyase activity, transferase activity, carbon-carbon lyase activity, cofactor binding, catalytic activity, and kinase activity were highly represented (**Figure 4C**; **Table S2**). Consistent with the results observed with TSWV, the KEGG pathway enrichment results also showed the overrepresentation of pathways relevant to essential metabolite biosynthesis, including carbon metabolism, photosynthesis, biosynthesis of secondary metabolites, biosynthesis of amino acids, fructose and mannose metabolism, autophagy, and starch and sucrose metabolism (**Figure 4D**). In contrast, the significantly enriched pathway relevant to resistance responses was autophagy (**Figure 4D**). Autophagy has been reported to be involved in plant defense responses to eliminate viral infections (Chiramel et al., 2013). Although HCRV did not affect the biosynthesis of JA, it triggered the activation of secondary metabolism in plants, implying its conserved function in the responses of plants to viral infection (**Figure 4D**). Additionally, HCRV also induced changes in the expression of genes relevant to ethylene synthesis, MAPK, WRKY, PR proteins, and proteolysis (**Figure 4B**). Overall, we found that the responses of plants to TSWV and HCRV infections were highly conserved, and the TSWV and HCRV infections were closely associated with the processes of JA synthesis and autophagy, respectively.

### The TSWV–HCRV complex synergistically infected plant tissue by suppressing plant basal resistance

**Figure 5:**
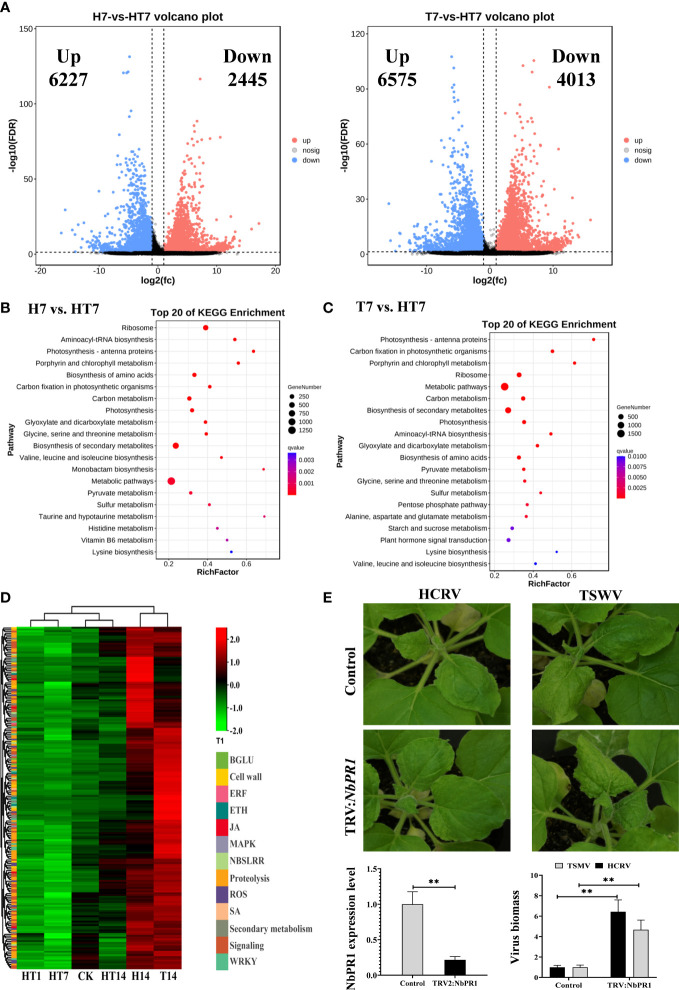
TH complex infection dramatically suppressed the genes relevant to plant resistance to biotic stress. **(A)** Volcano plots display the pattern of expression of *Nicotiana benthamiana* in TH vs. H (left) and TH vs. T (right) at 7 days post-inoculation. **(B)** Scatter plot of the enriched TOP 20 KEGG pathways for the DEGs of *N. benthamiana* at 7 days post-inoculation of the TH complex. **(C)** KEGG pathway enrichment in TH7 vs. T7 comparison. **(D)** A heat map showing the expression of genes relevant to plant resistance in *N. benthamiana* following the H virus, T virus, and TH complex challenges. The color intensity displayed in the heatmap is the mean TPM value of each gene and is used to exhibit the gene expression level. **(E)** Silencing of *NbPR1* results in a loss of resistance in tobacco to HCRV and TSWV. The silencing efficiency of *NbPR1* in tobacco through the VIGS system is detected using RT-qPCR. The biomass of both HCRV and TSWV in tobacco with *NbPR1* silencing is detected using RT-qPCR. **P < 0.01.

Because the main changes between the TH vs. T and TH vs. H comparisons were at 7 dpi of infection, we further analyzed the transcriptome profiles of tobacco at 7 dpi of HCRV, TSWV, and TH complex infections. In this study, we identified 6,227 upregulated genes and 2,445 downregulated genes in the H7 vs. TH7 comparison, whereas the T7 vs. TH7 comparison had 6,575 upregulated genes and 4,013 downregulated genes (**Figure 5A**). We then analyzed these DEGs from both pairwise comparisons by KEGG pathway and GO enrichment analyses. A functional analysis showed that both sets of DEGs from the TH vs. H and TH vs. T comparisons were enriched in 9,542 and 10,133 GO terms, respectively (**Tables S3**, **S4**). The results of GO enrichment on the DEGs from the H7 vs. TH7 comparison showed that these genes were highly enriched for several processes, including rRNA binding, lyase activity, peptide biosynthetic process, photosynthesis, carboxylic acid metabolic process, plastid, chloroplast, and ribosome (**Table S3**). Moreover, both enriched GO results from TH7 vs. T7 highly overlapped with those from the TH14 vs. H14 comparison (**Tables S3**, **S4**). GO enrichment analysis on the DEGs from the T7 vs. TH7 comparison displayed the overrepresentation of plastid, chloroplast, photosynthesis, carboxylic acid metabolic process, catalytic activity, isomerase activity, and lyase activity (**Table S3**). Both results suggested that complex infections with HCRV and TSWV could primarily alter plant photosynthesis and metabolism for essential metabolites and nutrients.

Subsequently, a pathway analysis was performed to predict the significantly enriched pathways that involved the DEGs from TH7 vs. T7 and TH7 vs. H7 comparisons to investigate the differences between individual infection of HCRV or TSWV and the TH complex infection. The significantly enriched pathways in the TH7 vs. T7 comparison were photosynthesis, ribosome, carbon metabolism, biosynthesis of secondary metabolites, biosynthesis of amino acids, starch and sucrose metabolism, and plant hormone signal transduction (**Figure 5C**). Moreover, the data from the TH7 vs. H7 comparison showed that the differences were primarily generated in the ribosome, photosynthesis, biosynthesis of amino acids, carbon metabolism, and biosynthesis of secondary metabolites (**Figure 5B**). Numerous pathways of essential metabolism were affected by TH infection at 7 dpi compared with neither HCRV nor TSWV (**Figures 5B, C**). Remarkably, some of these pathways are known to play an important role in plant defense responses to HCRV or TSWV infections, particularly plant hormone signal transduction and biosynthesis of secondary metabolites (**Figures 5B, C**). Importantly, we noted that many of the genes involved in plant basal resistance, particularly JA biosynthesis, MAPK, secondary metabolism, and pathogenesis-related proteins, could not be activated by TH complex infection but were triggered to upregulate under HCRV and TSWV infections (**Figure 5D**). Among pathogenesis-related proteins, we identified significant downregulation of *NbPR1*, *NbPR4*, and *NbPR5* in tobacco under TH complex infection compared to individual virus infection (**Figure 2D**). Then, we constructed tobacco with the silencing of these PRs to investigate their involvement in tobacco resistance to HCRV or TSWV. RT-qPCR assay confirmed the silencing of these genes in representative tobacco mutants (**Figure 5E**). Typically, *NbPR1* expression was suppressed by about 79% in tobacco compared to the controls (**Figure 5E**). Then, HCRV or TSWV were inoculated on these tobaccos. Fourteen days post-inoculation, we found observable symptoms caused by HCRV or TSWV on tobacco leaves compared to the controls (**Figure 5E**). Then, we determined the biomass of HCRV or TSWV in related tobacco, and the results showed that the biomass of HCRV or TSWV in tobacco with *NbPR1* silencing was 6.4- and 4.7-fold higher than that in the controls (**Figure 5E**), suggesting that silencing of *NbPR1* resulted in loss of resistance in tobacco. Thus, we propose that the TH complex can synergistically infect plant tissue to escape or suppress the plant’s basal resistance for their successful infection.

### The TSWV–HCRV complex deployed miRNA to suppress the plant basal resistance for their successful infections

**Figure 6:**
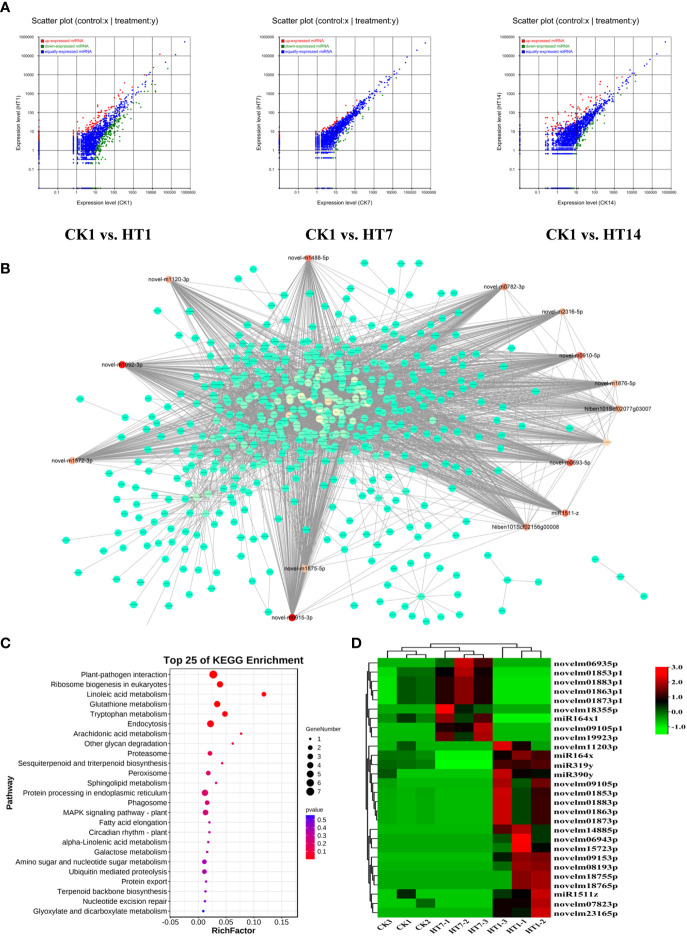
TH complex infection deployed various miRNAs of *Nicotiana benthamiana* to suppress the genes involved in plant resistance. **(A)** Scatter plots represent the differentially expressed miRNAs in CK vs. TH1, CK vs. TH7, and CK vs. TH14 pairwise comparisons. The up and downregulated miRNAs are shown in red and green, while the blue plots represent the miRNAs without significant changes. **(B)** A co-expression network of TH-triggered miRNA and genes relevant to plant resistance. The color represents the importance of each gene or miRNA in the network. The hub TH-triggered miRNAs are shown in red. **(C)** Top 20 enriched KEGG pathways based on the genes involved in the co-expression network. **(D)** A heatmap displays the dynamic changes of hub miRNAs in *N. benthamiana* following TH complex infection at 7 and 14 days. Heatmaps were generated using the mean TPM value of miRNAs in each treatment at different conditions. **P < 0.01.

MicroRNAs have been found to play important roles in plant–virus interactions, and they could interact with their target genes to degrade related mRNA or suppress its translation to regulate the series of plant responses (Ding, 2010; Finnegan and Pasquinelli, 2013; Borges and Martienssen, 2015). In this study, we further detected the dynamic expression variations of the miRNAs of tobacco following HT complex infection at 1, 7, and 14 dpi (**Figure 6A**). A total of 2,952 miRNAs were identified from all the sample libraries. In total, 517, 132, and 222 miRNAs were differentially expressed in the TH1 vs. CK, TH7 vs. CK, and TH14 vs. CK comparisons, respectively (**Figure 6A**). In addition, we found that a total of 346 miRNAs were upregulated in tobacco at 1, 7, and 14 dpi of TH infection, suggesting their involvement in plant–TH communication during the development of TH infection (**Figures 6A, D**). We then constructed the co-expression network of these 346 miRNAs with all the mRNAs of tobacco (**Figure 6B**). In addition, all the negative relationships between miRNA and mRNA were used to construct the topological network as shown in **Figure 6B**. Remarkably, 28 miRNAs were identified as hub regulators in this topological network, including 19 upregulated miRNAs at 1 dpi of TH infection and nine miRNAs at 7 dpi of TH infection (**Figure 6B**; **Table S4**). We then collected all the genes that negatively correlated with these miRNAs in the network and performed KEGG pathway enrichment analysis on these genes (**Figure 6C**). The results showed that these genes are primarily involved in plant–pathogen interaction, terpenoid biosynthesis, phagosomes, MAPK signaling, alpha-linolenic acid metabolism, and plant hormone signal transduction (**Figure 6C**). The most significant pathways were plant–pathogen interaction, MAPK signaling, linoleic acid metabolism, and metabolisms for secondary metabolites (**Figure 6C**), and these pathways that were significantly enriched were resistance-related processes associated with HCRV and TSWV infection, suggesting that the complex could trigger miRNAs to suppress plant basal resistance. Among these miRNAs, we noted that they exhibited two patterns of expression. A total of 19 miRNAs were highly expressed in tobacco at 1 dpi of TH complex infection, while nine miRNAs were primarily upregulated in TH-challenged tobacco at 7 dpi (**Figure 6D**).

### miRNA triggered by TSWV–HCRV complex directly binds to the promoters of resistance genes and suppresses their expression

**Figure 7:**
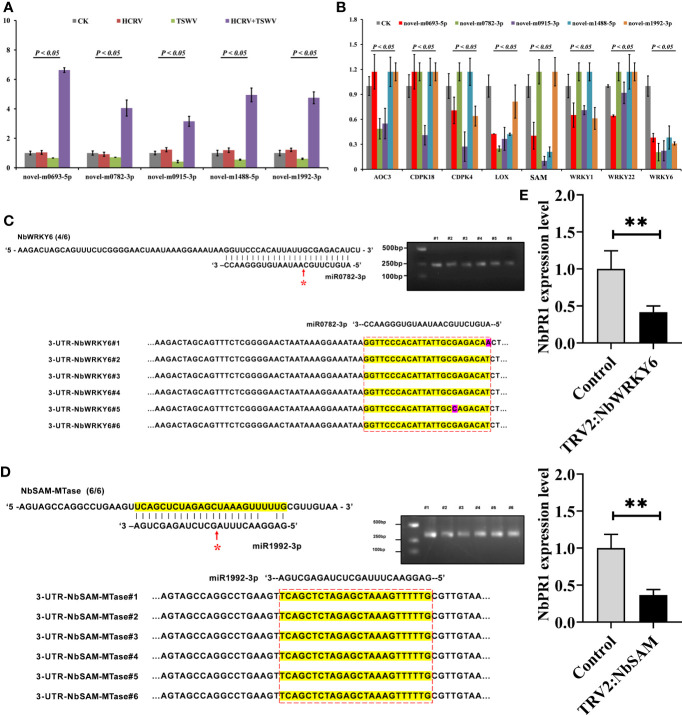
miRNA triggered by the TH complex directly binds to the promoters of NbSAM and NbWRKY6 to suppress the NbPR1-related resistance in tobacco. **(A)** The expression levels of the top five miRNAs from the hub network are detected using RT-qPCR. **(B)** RT-qPCR assay detects the expression levels of resistance genes triggered by individual viruses in tobacco with overexpression of hub miRNAs. **(C,D)** Novel-m0782-3p and miR1992-3p cleavage sites in *NbWRKY6* and *NBSAM* mRNAs validated by 5’ RLM-RACE, respectively. **(E)** RT-qPCR determines the expression level of NbPR1 in tobacco with the silencing of individual *NbSAM* and *NbWRKY6*. **P < 0.01.

To investigate the effect of these miRNAs associated with TH complex infection on mediating plant resistance, we overexpressed the top five miRNAs from the network, including novel-m0693-5p, novel-m0782-3p, novel-m0915-3p, novel-m1488-5p, and novel-m1992-3p in tobacco. Consistently, RT-qPCR assay confirmed that the TH complex triggered the upregulation of these five miRNAs, while individual HCRV or TSWV did not affect their expression or even suppress their expression in tobacco during the infection process (**Figure 7A**). Then, the expression levels of resistance genes that were triggered by infection with individual HCRV or TSWV viruses were further detected using RT-qPCR. The results showed that these resistance genes relevant to JA-, calcium-, and WRKY-mediated defense were dramatically inhibited by overexpression of these miRNAs (**Figure 7B**). Typically, all these miRNAs significantly inhibited the expression of *LOX6*, while novel-m0782-3p and novel-m0915-3p inhibited *AOC* expression in tobacco (**Figure 7B**), which could further suppress JA biosynthesis and related defense responses to reduce resistance in tobacco to virus. For calcium-related genes, individual novel-m0693-5p, novel-m0915-3p, or novel-m1992-3p overexpression mainly inhibited the expression of *CPK18* and *CPK4* that are involved in regulating calcium-related defense responses (**Figure 7B**). Additionally, all these miRNAs inhibited the expression of *WRKY6*, while novel-m0693-5p, novel-m0915-3p, or novel-m1992-3p overexpression also inhibited *WRKY1* and *WRKY22 in vivo* (**Figure 7B**). Additionally, miR1992-3p significantly inhibited the expression of *NbSAM in vivo* (**Figure 7B**). Taken together, these results suggest the involvement of these 28 miRNAs, especially novel-m0693-5p, novel-m0782-3p, novel-m0915-3p, novel-m1488-5p, and novel-m1992-3p, in the early communication between the plant and virus, which could further suppress the host defense responses so that both HCRV and TSWV could successfully infect the plant.

It is well known that miRNAs exert their functions by inhibiting the expression of target genes, and miRNAs need to be strictly complementary with their target genes to cleave at the pairing sites (Zhao et al., 2015). To reveal the target of these miRNAs, we predicted their potential targets through the website psRNATarget (http://plantgrn.noble.org/psRNATarget/). Under relatively strict parameters, *NbWRKY6* and *NBSAM* were predicted as the putative targets of novel-m0782-3p and miR1992-3p, respectively. Of these predicted target genes, we used 5’ RLM-RACE to validate the miRNA cleavage sites in target mRNAs. We found cleavage sites for novel-m0782-3p and miR1992-3p in *NbWRKY6* and *NBSAM* (**Figures 7C, D**), which supports the view that these genes are the direct targets of the corresponding miRNAs in tobacco. Among these target genes, a cleavage site for novel-m0782-3p was detected in the predicted miRNA complementary region, while for miR1992-3p, cleavage sites were detected in the predicted miRNA complementary region and upstream of this region (**Figure 7B**). Although *NbPR1* was not directly cleaved by novel-m0782-3p and miR1992-3p, we found that silencing of *NbWRKY* and *NbSAM* could result in downregulation of *NbPR1* (**Figure 7E**), suggesting that *NbWRKY* and *NbSAM* may be involved in regulating NbPR1. Overall, these results suggested that the TH complex could trigger novel-m0782-3p and miR1992-3p to suppress *NbWRKY6* and *NBSAM* expression, leading to downregulation of *NbPR1* and decreases in tobacco resistance to both viruses.

## Discussion

As sessile organisms, plants are unable to evade disadvantaged circumstances, especially biotic stress. Plants facilitate an innate immune system that can assist them in recognizing and defending against most pathogen attacks. However, a few highly evolved pathogens utilize a complex infection strategy to surmount plant resistance. Typically, cases of complex viral combinations have been extensively reported. In this study, we found that the TH complex could more easily infect tobacco plants and produce more severe symptoms than when infected with a single virus. A subsequent full-length transcriptome analysis suggested that the TH complex significantly compromised the basal resistance of tobacco plants, including the interruption of JA biosynthesis, inactivation of the MAPK cascade pathway, downregulation of genes relevant to secondary metabolisms, and activation of miRNA promotion, rendering the plants susceptible. Differently, single-inoculated HCRV or TSWV primarily trigger these responses at the transcription level, respectively. Importantly, we found that *NbPR1* was suppressed by the TH complex, leading to a loss of resistance to both viruses. The TH complex could trigger the upregulation of novel-m0782-3p and miR1992-3p to directly cut *NbWRKY6* and *NbSAM*, leading to downregulation of *NbPR1* and loss of resistance in tobacco to both viruses. These results suggest that HCRV and TSWV delicately operate to colonize tobacco in a mutually beneficial manner.

In general, viral infections trigger the systematic resistance of plants. Our study indicated that inoculation with either the H or T virus induced dramatic defense responses, particularly the upregulation of immunity-related genes such as MAPK, WRKY, and PRs. Additionally, genes associated with cell wall formation were downregulated, and even photosynthesis and carbohydrate metabolism were affected by virus infection. Despite the quick activation of immune events, evasion by the virus also made a tremendous difference in nutrient metabolism in tobacco. Consistently, mixed infection with TH also induced substantial changes in plant basal metabolism. Considering that immune events are energy-intensive, the plant redistributes energy to manage an unpredictable situation (Wang et al., 2021). In addition, viruses need to use nutrients from host plants to replicate and then mislead the plants to consume more energy (Sanfaçon, 2015). Strikingly, mixed infection with TH seemed to avoid triggering the immune system to express basal resistance genes. It has been found that mixed infections lead to the genetic recombination of viruses, resulting in new viruses with enhanced virulence and a wide host range (Gil-Salas et al., 2011). We therefore reasoned that the innate immune system might not recognize mixed viruses and, therefore, could not activate defense responses. Our study showed that infection with a single T virus triggered the biosynthesis of JA, whereas the H virus could not. JA is an important molecule to trigger immune events, such as the expression of PR genes, biosynthesis of phytoalexins, and enhanced crosstalk with other immune pathways (Ruan et al., 2019). Thus, we deduced that the H virus could help the T virus escape JA-related defense responses. For the H virus, the resistance genes involved in autophagy were markedly induced in tobacco but not in T-infected plants. Plants quickly prime the autophagy system to eliminate microorganisms to protect themselves from viruses. Overall, we concluded that the virus complex has more advantages in escaping the plant immune system than an individual virus.

It is worth noting that variation in miRNA was closely implicated in the suppression of basal resistance during TH co-infection. Further co-expression network analysis showed that numerous mRNAs are involved in suppressing MAPK signaling transduction, the defense hormone signaling pathway, and the biosynthesis of secondary metabolites. A total of 28 key miRNAs could play essential roles in binding and suppressing the translation of resistance genes during plant–virus confrontation at the preliminary stages. Overall, we summarized that co-infection with viruses mainly affected the regulatory amplitude or direction of some pivotal genes in the basal resistance of plants. Typically, co-infection with viruses could manipulate the expression of endogenous miRNA in plants to suppress genes involved in plant basal resistance. These results are expected to shed light on the mechanism of co-infection between HCRV and TSWV, which provides theoretical support for the field management of co-infecting viruses.

## Data availability statement

The original contributions presented in the study are publicly available. This data can be found here: NCBI (https://www.ncbi.nlm.nih.gov/), accession PRJNA945175.

## Author contributions

MG and HH designed the experiments and wrote the original draft of this manuscript and revision. ZJ, XG, and HT performed the experiments and analyzed the data. YZL and YTL developed the research concept and managed the funding for the publication. All authors contributed to the article and approved the submitted version.
